# Synchronous primary gallbladder squamous cell carcinoma and colon adenocarcinoma: A case report and literature review

**DOI:** 10.1097/MD.0000000000045857

**Published:** 2025-11-07

**Authors:** Min-Ho Shin, Young-Hun Kim, Sung-Chul Lim

**Affiliations:** a Department of Surgery, School of Medicine, Chosun University, Gwangju, Korea; b Department of Pathology, School of Medicine, Chosun University, Gwangju, Korea.

**Keywords:** adenocarcinoma, colon, gallbladder, squamous cell carcinoma

## Abstract

**Rationale:**

Gallbladder (GB) malignancy is the most common biliary malignancy; however, squamous cell carcinoma of the gallbladder (GBSCC) is extremely rare. Synchronous primary GB and colon cancers are very rare, but they are still adenocarcinomas.

**Patient concerns:**

The patient was a 68-year-old male who had symptoms of anemia and underwent a comprehensive examination. Irregular thickening of the GB wall and localized bowel wall thickening in the ascending colon were observed.

**Diagnoses:**

Colonoscopy revealed a large mass in the ascending colon, and adenocarcinoma was diagnosed by biopsy.

**Interventions:**

Right hemicolectomy and extended cholecystectomy were implemented.

**Outcomes:**

The patient was diagnosed with synchronous primary pure GBSCC and colon adenocarcinoma. Anemia was found on the 10th postoperative day, and syncope occurred on the 12th day; computed tomography showed a gastroduodenal artery aneurysm, and covered stent insertion was attempted but failed. Consequently, intimal dissection and common hepatic artery embolization resulted, leading to hepatic failure, and the patient died on the 18th day.

**Lessons:**

We report a case of synchronous primary pure GBSCC and colon adenocarcinoma. Synchronous primary GB and colon adenocarcinomas have rarely been reported. However, synchronous primary pure GBSCC and colon adenocarcinomas have not yet been reported in English literature. Here, we present a literature review on the pathogenesis of GBSCC and synchronous GB and colon cancer.

## 1. Introduction

Multiple primary cancer (MPC) is a rare disease with a low incidence rate; however, its frequency has recently been increasing. MPC requires each tumor to be histopathologically confirmed; each tumor must be geographically separated and distinguished, and separated by a normal mucosa, and the probability that one tumor is a metastasis to another should be excluded.^[1]^

Although the pathogenesis of MPCs has not been clearly identified, it can be divided into host-related factors, lifestyle factors, and environmental influences, depending on the etiological factors.^[2]^

If a second malignancy occurs at the same time as the first malignancy or within 6 months in the same person, it is called synchronous cancer; in other cases, it is called metachronous cancer.^[3]^

Synchronous cancer is very rare and is often misdiagnosed, and there is no international consensus regarding its diagnosis and treatment.^[4]^

The most common combinations of synchronous cancer are esophageal cancer and gastric cancer, gastric cancer and lung cancer, and bladder cancer and prostate cancer in order.^[5]^

Synchronous primary gallbladder (GB) carcinoma and colon adenocarcinoma are very rare, and only 9 cases have been reported in the English literature, all GB carcinomas are 8 adenocarcinomas and 1 neuroendocrine carcinoma.^[6]^

GB stones and chronic inflammation are the most common risk factors for GB cancer. A high body mass index (BMI) contributes to the formation of gallstones, which are a well-known risk factor for GB cancer.^[7]^

Patients diagnosed with GB cancer are significantly more likely to develop additional primary gastrointestinal cancers, which can lead to secondary cancers of the stomach, small intestine, large intestine, liver, pancreas, and bile ducts.^[8,9]^

Based on synchronous primary pure squamous cell carcinoma of the gallbladder (GBSCC) and colon adenocarcinoma cases that have not been reported to date, we aimed to determine the pathogenesis of GBSCC through a literature review and investigate the pathogenetic links between GBSCC and colon adenocarcinoma.

## 2. Case report

A 68-year-old man underwent a comprehensive examination for anemia at a local clinic. Abdominopelvic computed tomography revealed localized thickening of the bowel wall in the ascending colon, and colon cancer was suspected. In addition, irregular thickening of the GB wall was observed, which invaded the adjacent liver; therefore, primary GB cancer was suspected rather than metastatic lesions.The patient was transferred to our hospital for surgical measures. At the time of admission, the patient’s blood pressure was 135/63 mm Hg and the BMI was 18.9. He had no history of diabetes, smoking or drinking. There was no other relevant patient medical and surgical history. Family history was not significant especially for any cancer. Blood tests showed hemoglobin 9.2 g/dl, hematocrit 29.1%, white blood cells 2960/µL, and platelets 159,000/µL. Macrocytic normochromic anemia has been identified.

On abdominopelvic computed tomography, a 51 mm-sized fungating mass was observed in the proximal ascending colon and cecum, and the surrounding lymph nodes were enlarged. In addition, a 36 mm-sized heterogeneous enhancing mass was observed in the GB and adjacent liver, with layer destruction of the GB wall. These findings were more suggestive of primary GB cancer than of metastatic lesions. Soft tissue lesions around the right hepatic and cystic arteries were suspected, which seemed to have the potential for tumor infiltration.

**Figure 1.**
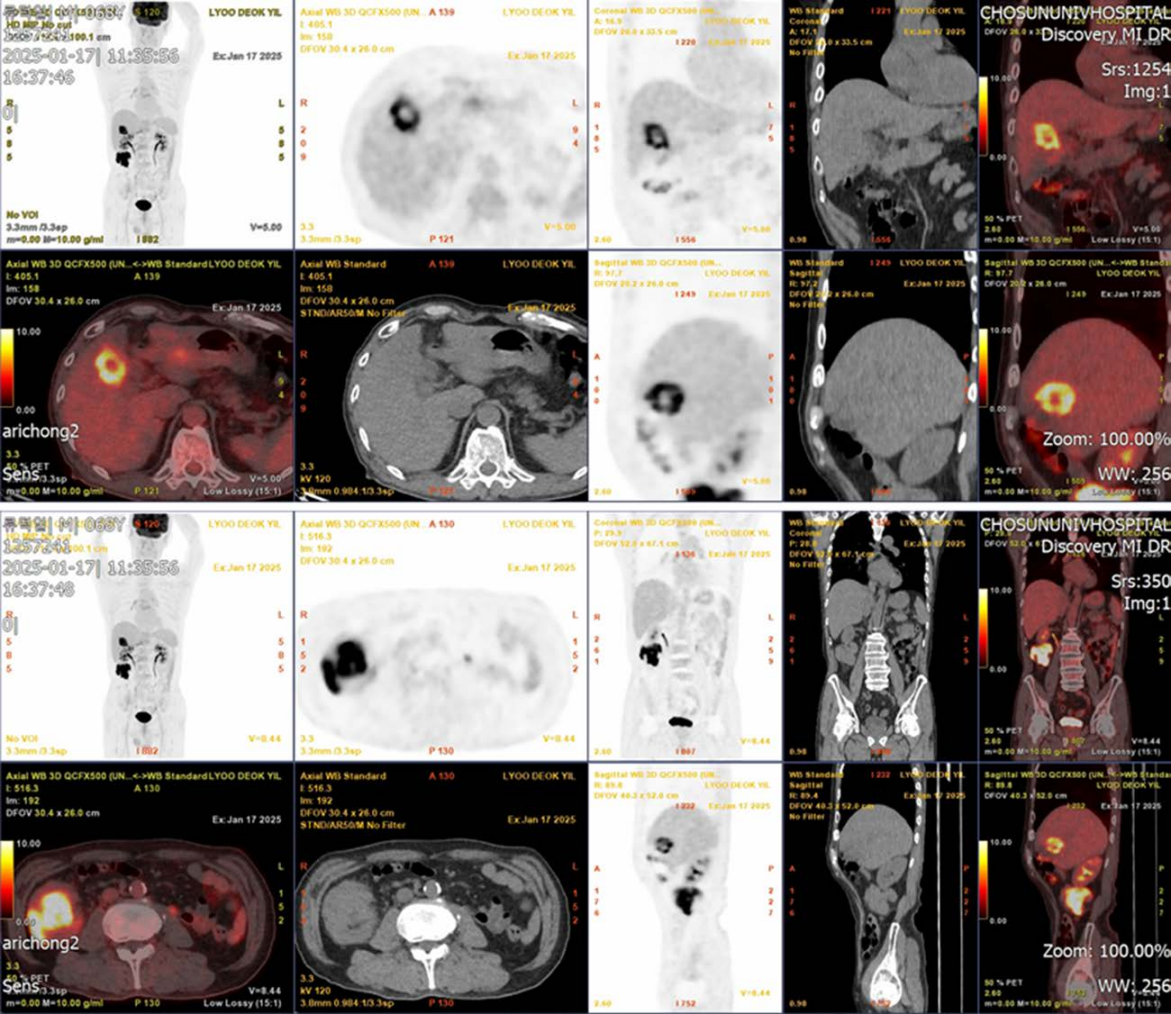
Preoperative imaging findings. F-18 FDG PET-CT shows hypermetabolism in approximately 6 cm-sized mass in the ascending colon, and in irregular mass involving the GB and adjacent liver.

A mass was identified in the ascending colon on colonoscopy, and adenocarcinoma was confirmed by biopsy. The ascending colon, GB, and adjacent liver showed hypermetabolic lesions on fluorine-18 fluorodeoxyglucose positron emission tomography (F-18 FDG PET)-CT performed before surgery. In addition, hypermetabolic lesions were not observed in other areas (Fig. 1). After right hemicolectomy, extended cholecystectomy was performed in turn.

**Figure 2.**
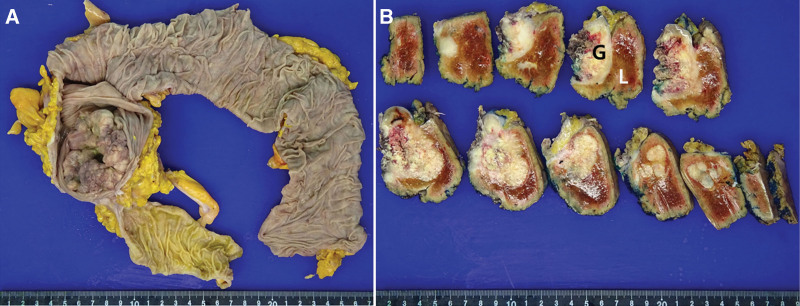
Gross findings of the colon and gallbladder tumors. Ulcerofungating cancer involving the proximal ascending colon and cecum is identified (A). Serial section of the gallbladder and adjacent liver shows massive extension of the gallbladder tumor (G) into the liver (L) parenchyma (B).

A 6.3 × 5.4 cm-sized ulcerofungating mass was observed in the proximal ascending colon and cecum. A mass measuring 5.5 × 3.9 cm in size was observed on the GB wall, which was extended to the adjacent hepatic parenchyma (Fig. 2).

**Figure 3.**
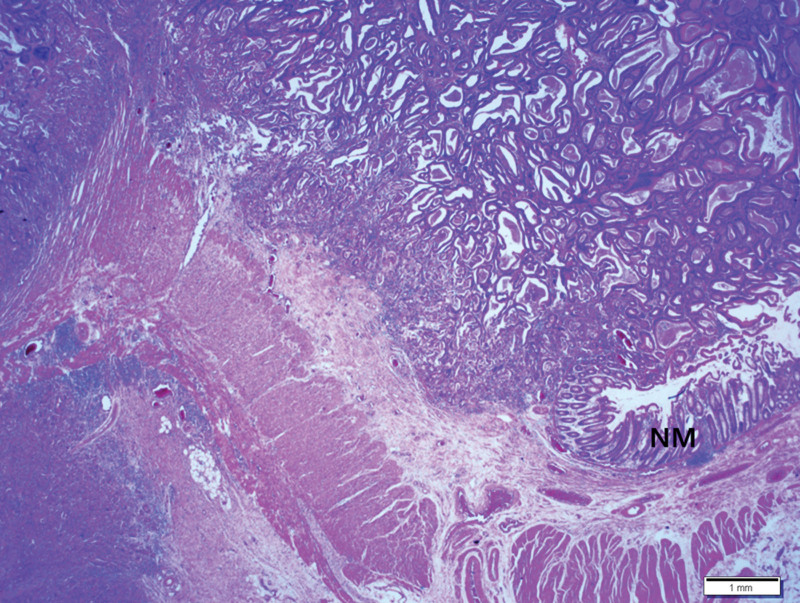
Histopathological findings of the colon. Ulcero-fungating and -infiltrative adenocarcinoma is identified from the mucosa to subserosa. NM: adjacent normal mucosa. Scale bar measures 1 mm.

Histopathological examination revealed a moderately differentiated adenocarcinoma in the colon that extended to the subserosa but did not show lymph node metastasis (Fig. 3). In immunohistochemical staining using colon cancer tissue, MLH2 and MSH6 showed positive reaction in the nucleus, but MLH1 and PMS2 were negative. However, as a result of performing next-generation sequencing using surgical samples, no genetic mutations including mismatch repair (MMR) genes (*MLH1, MSH2, MSH6,* and *PMS2*) and *BRCA* were found.

**Figure 4.**
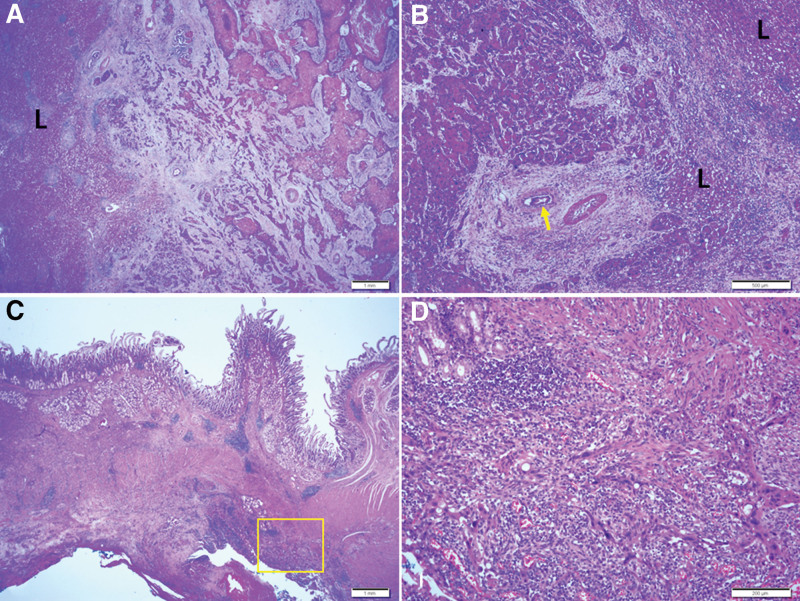
Histopathological findings of the liver and duodenum. Low magnification of the liver shows direct extension of squamous cell carcinoma in liver parenchyma (A). Higher power view of the liver shows squamous cell carcinoma in the deep portion. Arrow indicates bile duct (B). Duodenum shows infiltrating squamous cell carcinoma in the serosa to proper muscle (C). Higher power view of the yellow boxed area of Figure C shows squamous cell carcinoma in the subserosa and muscle layer (D). L: liver parenchyma. Scale bars measure A: 1 mm, B: 500 µm, C: 1 mm, and D: 200 µm.

In addition, pure squamous cell carcinoma (SCC) was diagnosed in the GB, which extended to the adjacent liver and invaded the muscle proper in the adjacent duodenum. Perineural invasion was observed; however, no lymph node metastasis was detected. Chronic acalculous cholecystitis was also observed (Fig. 4).

**Figure 5.**
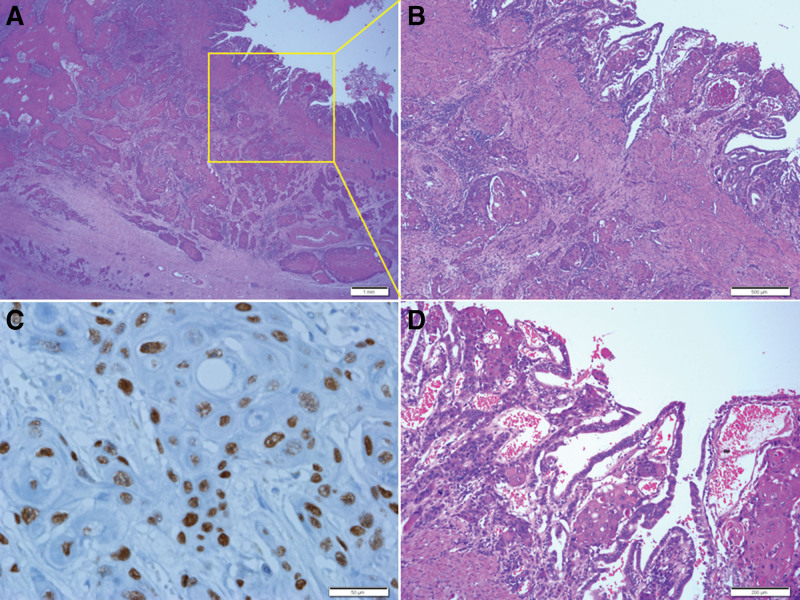
Histopathological findings of the gallbladder tumor. Transmural squamous cell carcinoma in the gallbladder wall (A). Higher power view of the yellow boxed area of Figure A shows squamous cell carcinoma from the mucosa to adventitia (B). Immunohistochemical staining for p40 shows positive nuclear immunoreactivity in the tumor cells (C). Higher magnification of mucosa in Figure B shows well- and moderately differentiated squamous cell carcinoma (D). Scale bars measure A: 1 mm, B: 500 µm, C: 50 µm, and D: 200 µm.

Frequent foci of squamous metaplasia were observed in the GB mucosa, and cytologic atypia of columnar cells was observed in adjacent areas. They were abruptly converted into well- or moderately differentiated SCC (Fig. 5).

**Figure 6.**
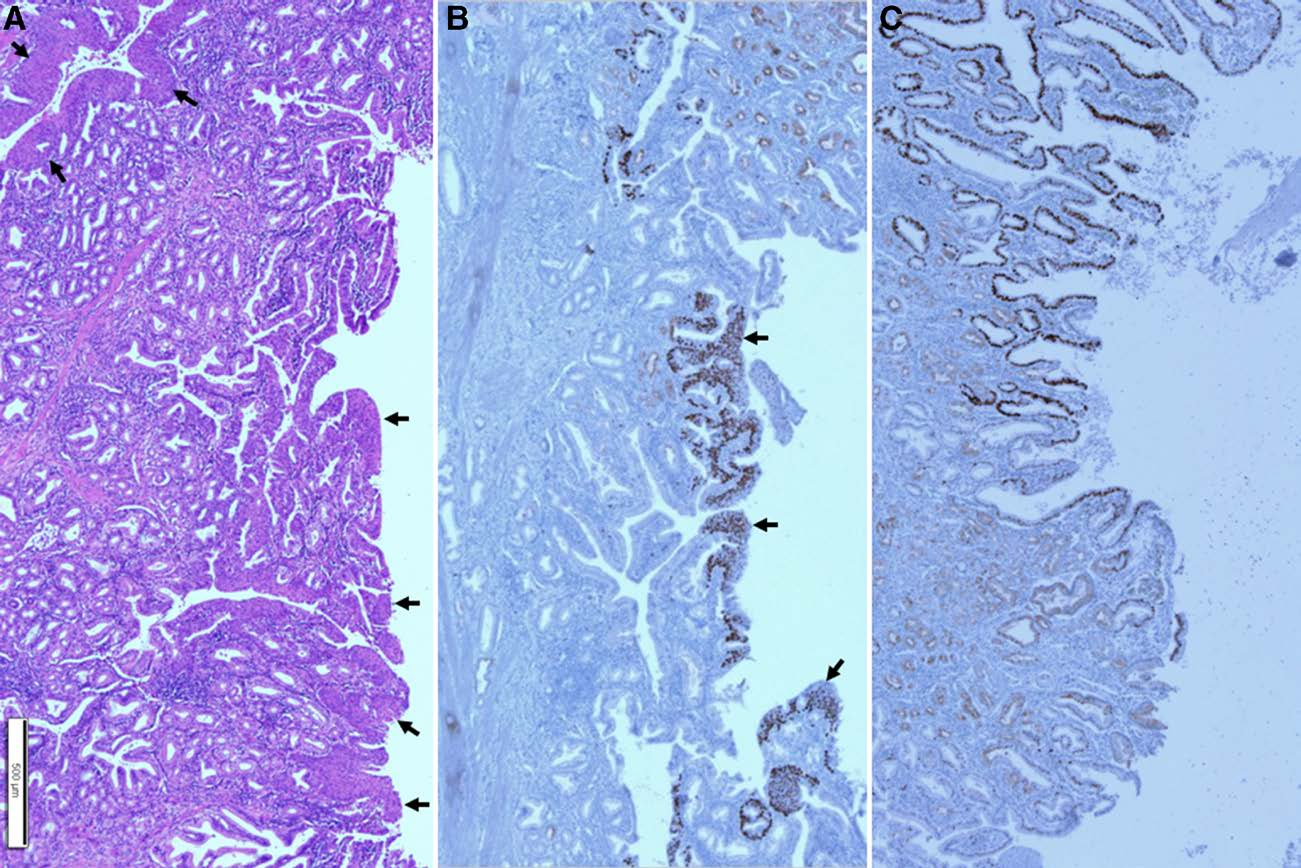
Histopathological findings of the gallbladder mucosa. Mucosal lining shows abrupt squamous metaplasia (arrows) with chronic inflammation (A). Immunohistochemical staining for p40 shows abrupt positive nuclear staining (arrows) in the squamous metaplasia (B). Immunohistochemical staining for p53 protein shows positive nuclear immunoreactivity in the area of atypical columnar linings (upper 2/3) but negative immunoreactivity in the area of normal-looking mucosal linings (lower 1/3) (C). Scale bar measures 500 µm.

Squamous metaplasia was caused by abruption and was positive for p40 immunohistochemical staining. The atypia of columnar cells observed in adjacent areas was positive for p53 protein on immunohistochemical staining. In addition, a large part of the area where atypia of the columnar cells was observed showed positive findings at p40; therefore, some of these were considered squamous metaplasia with atypia (Fig. 6). Several measures were taken to rule out the possibility of adenocarcinoma. First, paraffin blocks were produced for all tumors of the GB mucosa identified by the eyes to confirm whether adenocarcinoma coexisted, and if there were suspicious areas, serial sections were performed and carefully examined. Second, the suspicious or ambiguous areas were excluded through p40 immunostaining. But no areas that could be considered as adenocarcinoma were observed, except for the atypia of the columnar cells. So, the patient was diagnosed with pure SCC.

**Figure 7.**
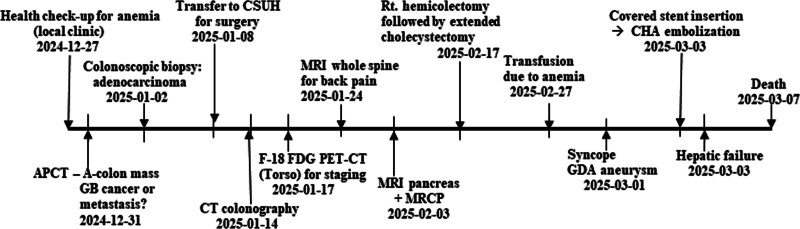
Chronological summary of the patient. APCT = abdominopelvic computed tomography, A-colon = ascending colon, CHA = common hepatic artery, CSUH = Chosun University Hospital, MRCP = magnetic resonance cholangiopancreatography, GB = gallbladder, GDA = gastroduodenal artery.

The patient was confirmed to have anemia on the 10th postoperative day and underwent several transfusions. On the 12th day after surgery, there was a syncope with reduced blood pressure, loss of consciousness, and the drainage tube changed to bloody. CT was performed as an evaluation, where a new intraperitoneal hematoma and gastroduodenal artery (GDA) aneurysm were found. Angiogram was performed as a first aid, and it can usually be solved by inserting a covered stent, but the stent insertion failed due to a radiologic technical failure. In this process, the related blood vessels were damaged, and the bleeding was not controlled. In the end, we had no choice but to choose to block the entire common hepatic artery (CHA) through CHA embolization. Hepatic artery blood flow was blocked through CHA embolization, resulting in a hepatic failure. Eventually the patient died on the 18th postoperative day. Blood tests on the day of death were hemoglobin 6.6 g/dl, hematocrit 20.9%, leukocytes 2810/µL, and platelets 26,000/µL. The main events from hospitalization to death of the patient are summarized in Figure 7 according to the passage of time, and the changes in liver function test are summarized in Table 1.

**Table 1:** Summary of liver function test change over time.

Date Parameter	Preop. check 2025-02-10	2025-03-01	2025-03-02	2025-03-03	2025-03-04	2025-03-05	2025-03-06	Death 2025-03-07	Normal value
AST	20	19	85	9762	5532	1381	667	415	5–40 U/L
ALT	18	29	57	4762	2961	1523	1013	460	5–40 U/L
TB	0.44	0.72	2.82	3.90	14.6	18.1	17.6	13.7	0.2–1.2 mg/dL
Lipase								104	13–55 U/L

ALT = alanine transaminase, AST = aspartate transaminase, TB = total bilirubin.

## 3. Discussion

The incidence of MPCs is on a steady rise, reportedly 0.7 to 11.7%, and most occur in the respiratory, gastrointestinal, or genitourinary system. The development of diagnostic technologies has had the greatest impact on this increase pattern.^[10]^ Complex risk factors are involved in the pathogenesis of MPCs, including genetic predisposition, environmental exposure, host factors, primary cancer treatments, and immunosuppressive conditions.^[11]^ Among these, genetic predisposition is the most pivotal, including inherited cancer syndromes such as Lynch syndrome, mutations in DNA repair genes, and breast cancer susceptibility gene (*BRCA*) mutation.^[12,13]^ However, in this case, there was no *MMR* or *BRCA* mutation.

Environmental exposure and host factors are also important, such as smoking, alcohol consumption, exposure to ionizing radiation, chemicals, obesity, and hormone therapy.^[14]^

Understanding these risk factors is crucial for the prevention, early diagnosis, and treatment of MPCs.

According to one report, cholelithiasis is associated with female breast cancer, colon cancer, pancreatic cancer, and small intestine cancer,^[15]^ and synchronous breast and GB lesions are common in women; therefore, if one lesion is found, the clinician should make an effort to find another.^[16]^

GB cancer is more common in women and usually occurs in women over the age of 65 years, with an average age of 75 years.^[17]^ The most common risk factors for GB cancer are cholelithiasis and cholecystitis; a high BMI contributes to the development of cholelithiasis, which is a well-known risk factor for GB cancer.^[7,18,19]^ The overall incidence of GB cancer in patients with cholelithiasis is reported to be 0.5 to 1.5%, and cholelithiasis is present in 70% to 90% of patients with GB cancer.^[20,21]^

However, the present case involved a male with normal weight, no family history of cancer or diabetes, no drinking or smoking habits, and cholecystitis without stones.

Patients diagnosed with GB cancer have a significantly increased risk of developing additional primary gastrointestinal cancers, including cancers of the stomach, small and large intestines, liver, pancreas, and bile ducts.^[8,9]^ Most patients who developed synchronous primary carcinoma in the GB and colorectum were women (seven of 9).^[6]^ In the patients with GB carcinoma who developed synchronous primary cancer in places other than the colorectum, 7 had cholelithiasis or cholecystitis (one case was a cholesterol polyp and the other was not mentioned) (Table 2).^[4,6,22,23]^

**Table 2:** Clinical summary of the reported synchronous primary carcinoma of the gallbladder and colorectum.

Case No.	Age (yr)	Sex	Location	Diagnosis	TNM (stage)	Combined/underlying disease (GB)	Remarks
1	79	F	TC	MAC, MD	pT3, N0, M0 (IIA)	Chronic calculous cholecystitis	
			GB	AC, WD	pT2, N0, M0 (II)		
2	70	M	SC	AC, WD	(I)	NC	Arteriosclerosis MSI (+)
			GB	PAC	(I)		
			Stomach	AC, WD	pT1, N0, M0 (IIA)		
3	72	F	R	AC, MD	pT2, N0, M0 (II)	Chronic calculous cholecystitis	
			GB	AC, MD	pT2, N0, M0 (II)		
4	40	F	R	AC, MD	pT3, N0, M0 (IIA)	Chronic calculous cholecystitis	Normal MMR genes
			GB	AC, MD	pT2, N0, M0 (II)		
5	30	F	SC	AC, MD	pT4b, N1, M0 (IIIC)	Chronic calculous cholecystitis	Nonsmoker non-alcoholic
			GB	AIS	Tis, N0, M0 (0)		
6	59	F	SC	AC, WD	pT3, N1, M0 (IIIB)	Cholesterolic polyp, GB	
			GB	NEC	LCHG (IIIB)		
7	75	F	SC	AC, MD	pT3, N2, M0 (IIIC)	Chronic calculous cholecystitis	DM hypertension hypercholesterolemia
			GB	AC, PD	pT2b, N1, M0 (IIIB)		
8	59	M	ASC	AC	pT1, N0, M0 (I)	Chronic calculous cholecystitis	DM
			GB	AC	pT3, N0, M0 (IIIA)		
9	48	F	TC	AC, WD	pT3, N0, M0 (IIA)	Chronic xanthogranulomatous cholecystitis	Intact nuclear MMR proteins
			GB	AC, MD	pT2b, N1, M0 (IIIB)		
Present case	68	M	ASC	AC, MD	pT3, N0, M0 (IIIA)	Chronic acalculous cholecystitis	Normal MMR genes nonsmoker non-alcoholic
			GB	SCC	pT3, N0, M0 (IIIA)		

AC = adenocarcinoma, AIS = adenocarcinoma in situ, ASC = ascending colon, DM = diabetes mellitus, F = female, GB = gallbladder, LCHG = large cell high grade, M = male, MD = moderately differentiated, MMR = mismatch repair, MSI = microsatellite instability, NC = non contributory, NEC = neuroendocrine carcinoma, PAC = papillary adenocarcinoma, PD = poorly differentiated, R = rectum, SC = sigmoid colon, SCC = squamous cell carcinoma, TC = transverse colon, WD = well differentiated.

Multiple molecular pathways such as hypoxia-inducible factors (HIF) and C-X-C motif chemokine receptor 4 (CXCR4) are involved in normal mucosal cells that are exposed to chronic inflammation and converted to cancer.^[24,25]^ Therefore, targeting hypoxia-related pathways such as CXCR4 or HIF signaling may be a promising method for GB cancer intervention.

GBSCC is believed to originate from the basal layer of the mucosal epithelium and result from squamous metaplasia or SCC differentiation of preexisting adenocarcinoma.^[26]^ GBSCC is particularly rare, accounting for 1% to 4% of all GB tumors, and almost all are adenocarcinoma.^[26–29]^ Compared with adenocarcinoma, GBSCC is characterized by larger lesions, older age at the time of expression, higher histological grade, and a more advanced pathological stage.^[30]^ GBSCC shows rapid proliferation, and because of its anatomical histological characteristics, early spread to adjacent and distant organs occurs; therefore, the prognosis is the worst among histological subtypes, with a median survival time of 7 months and a 5-year survival rate of <12%.^[31]^ Unfortunately, this patient died due to postoperative complications, but the prognosis is presumed to have been poor even if he recovered.

We believe that GDA aneurysm is caused by a dissection around hepatoduodenal ligament and CHA during lymph node harvest. To avoid complications such as in this case, first, the blood vessel should be dissected a little more meticulously during lymph node harvest, and second, it encourages the occurrence of aneurysm in the event of intraperitoneal infection or bile leakage, so prevention and early treatment are necessary.

In this case, chronic cholecystitis appeared to have occurred, followed by squamous metaplasia and SCC, which extended massively with an adjacent liver and invaded the duodenal muscle layer.

Patients with primary colorectal cancer are more likely to develop secondary primary cancers, and the incidence of secondary primary cancers increases in the colon and rectum.^[32]^ Colorectal cancer patients also have an increased risk of developing cancer in the small intestine, cervix, breast, kidney, thyroid, stomach, bladder, and respiratory system and melanoma but a decreased risk of GB cancer.^[6,8]^ Therefore, synchronous cancers of the GB and colon are rare.

## 4. Conclusion

Synchronous primary GB cancer and colon adenocarcinoma are very rare; thus, 9 cases have been reported so far. GB cancer occurs mostly in women and is associated with cholelithiasis or cholecystitis. However, this patient was male, had acalculous cholecystitis, and was diagnosed with pure GBSCC. Therefore, this is an extremely rare and potentially first reported case of synchronous primary GBSCC and colon adenocarcinoma reported in the English literature and is reported with a review of the literature in light of its rarity.

## Author contributions

**Conceptualization:** Sung-Chul Lim.

**Data curation:** Min-Ho Shin, Young-Hun Kim, Sung-Chul Lim.

**Funding acquisition:** Sung-Chul Lim.

**Methodology:** Min-Ho Shin, Young-Hun Kim, Sung-Chul Lim.

**Supervision:** Sung-Chul Lim.

**Validation:** Sung-Chul Lim.

**Writing – original draft:** Min-Ho Shin, Young-Hun Kim.

**Writing – review & editing:** Sung-Chul Lim.
